# Micro-Organisms without Leucocytes in Cerebro-Spinal Fluid

**Published:** 1909-02-20

**Authors:** 


### MICRO-ORGANISMS WITHOUT LEUCOCYTES IN CEREBRO-SPINAL FLUID.

It has been shown by Achard and Ramond that cerebro-spinal fluid may abound in micro-organisms and yet exhibit very few cells. For instance, Achard records that a man of thirty years of age came into hospital for symptoms of microbial infection, with pyrexia, severe headache, and retraction of the head. Cerebro-spinal meningitis being suspected, lumbar puncture was performed and clear fluid was obtained; films of the centrifugalised deposit showed no cell-elements, but large numbers of micro-organisms which did not retain the stain by Gram's method and morphologically resembled the bacillus coli communis. Believing that some accidental contamination of the films or apparatus had occurred, Achard repeated the lumbar puncture next day. The result was precisely the same as before: there were no cell-elements in the films, but numbers of bacilli. The patient made a perfect recovery.

He records another case in which the diagnosis was confirmed by autopsy. A woman, aged 32 years, was getting up on October 18 when she was suddenly seized with the most violent headache. She vomited her breakfast, took to her bed at mid-day, and vomited twice more before October 19. In the early morning of October 19 she became comatose, and was removed to hospital at mid-day. The slightest pinching or pricking of the skin caused her to cry out with pain, although she was unconscious. There was no paralysis. The pupils were equal, of medium size, and they reacted to light. There was photophobia. Kernig's sign was well marked, and there was rigidity of the back of the neck. The temperature was 103.6° F., the pulse-rate 65. The condition was much the same on October 20, except that there was now well-marked strabismus, and the temperature was 102.6° F. Lumbar puncture was performed, and 50 cubic centimetres of pale, almost clear fluid spurted out. Examined directly in films, it was found to contain large numbers of diplococci that retained the stain by Gram's method. Some idea of their abundance may be gathered when it is mentioned that the fluid resembled a laboratory culture in bouillon. Centrifugalisation did not render the fluid clear. The centrifugalised deposit contained only a quite small number of polymorphonuclear leucocytes, but a great abundance of diplococci.

The patient died the same night, and the post-mortem examination showed a suppurative meningitis over the brain with no lesions anywhere else. In view of these cases it is well to remember that paucity of cell-elements in cerebro-spinal fluid by no means implies that micro-organisms are not present.

